# Purification of Biodiesel Polluted by Copper Using an Activated Carbon Prepared from Spent Coffee Grounds: Adsorption Property Tailoring, Batch and Packed-Bed Studies

**DOI:** 10.3390/molecules30030483

**Published:** 2025-01-22

**Authors:** Daniel Eduardo Cárdenas-Piñeros, Hilda Elizabeth Reynel-Ávila, Lizbeth Liliana Díaz-Muñoz, Adrián Bonilla-Petriciolet, Carlos Javier Durán-Valle, Marta Adame-Pereira

**Affiliations:** 1 Tecnológico Nacional de México-Instituto Tecnológico de Aguascalientes, Aguascalientes 20256, Mexico; danielcardenasuis@gmail.com (D.E.C.-P.); betuli@yahoo.com (H.E.R.-Á.); lizbeth_liliana_3@hotmail.com (L.L.D.-M.); petriciolet@hotmail.com (A.B.-P.); 2 CONAHCYT—Consejo Nacional de Humanidades, Ciencias y Tecnologías, Ciudad de México 03940, Mexico; 3 Facultad de Ciencias Químicas, Universidad Autónoma de Nuevo León, Nuevo León 66455, Mexico; 4 IACYS—Instituto del Agua, Cambio Climático y Sostenibilidad, Universidad de Extremadura, 06006 Badajoz, Spain; martaap@unex.es

**Keywords:** biofuel purification, energy transition, heavy metal removal

## Abstract

Biodiesel produced via oil transesterification often contains metallic impurities, such as copper, which affects its quality and engine performance. This study explores the use of activated carbon prepared from spent coffee grounds to remove copper from biodiesel. Activated carbon samples were prepared via biomass pyrolysis and chemical activation with KOH and HNO_3_. The optimal conditions for copper adsorption were determined using a Taguchi L9 design. Maximum adsorption capacities were 13.4 and 17.3 mg/g at 30 and 40 °C, respectively, in batch adsorbers. In packed-bed columns, the axial dispersion reduced the adsorption efficiency obtaining bed adsorption capacities from 1.9 to 5.1 mg/g under tested experimental conditions. Adsorbent characterization and adsorption modeling indicated that copper removal was driven by multi-cationic interactions, where carboxylic groups from carbon surface acted as key active sites. The new adsorbent outperformed commercial bone char, making it a cost-effective alternative to improve biodiesel production contributing to the energy matrix diversification.

## 1. Introduction

In recent decades, the global energy demand has intensified due to continuous demographic growth, which implies satisfying basic needs and improving the quality of life of the population [1,2]. Environmental pollution and its associated impacts (e.g., glacier melting, biodiversity loss, and sea level rise) caused by greenhouse gas emissions have been attributed to the dependency on fossil energy sources, mainly in the transportation sector [3,4]. Although worldwide statistics indicate that the availability of petroleum-derived energy resources is continuously declining, fossil fuels are still the primary energy source for a wide spectrum of anthropogenic activities and will continue to contribute to the emission of greenhouse gases in forthcoming years. These factors have been the key drivers for the intensification of the international agenda of energy transition with the aim of developing sustainable alternatives to replace non-renewable fuels [3,5].

Biofuels prepared from residual biomass or living organisms (e.g., microalgae and plants) are a sustainable option to diversify the energy matrix of several countries and mitigate the environmental impacts caused by different industrial sectors including transportation [1,6,7]. One of the most studied biofuels is biodiesel, which is typically produced from renewable materials, such as vegetable oils and animal fats [6,8]. The properties of this biofuel are similar to those of diesel in terms of density, cetane number, efficiency, flash point, and engine performance. For this reason, blends of both fuels can be utilized, and in some cases, biodiesel can completely replace the traditional fuel [9]. Overall, the benefits of biodiesel over its counterpart (i.e., diesel) include its biodegradable, renewable, and non-toxic properties, along with a better combustion emission profile characterized by lower emissions of unburned hydrocarbons, particulate matter, and CO [8,10].

Biodiesel can be produced via transesterification of raw glyceride sources with alcohol (mainly methanol or ethanol) in the presence of a catalyst to accelerate the reaction and improve the yield [11]. Metal-based homogeneous and heterogeneous catalysts are used in this reactive system [12]. However, this production route has the main disadvantage that the utilization of metallic-based catalysts may produce biodiesel containing dissolved metals as impurities, which affects the engine performance and reduces its lifespan [13,14]. The presence of metals alters biodiesel quality, accelerating its degradation, reducing storage time, in addition to causing oxidation, corrosion, instability, clogging, sediment formation, improper combustion, and other issues that interfere with engine operation [15,16]. Note that the metals contained in biodiesel may eventually reach the atmosphere, polluting the air, via gas emissions during combustion [17]. The allowable concentrations of metals and other specifications to ensure biodiesel quality are regulated by international standards [18]. These regulations guarantee that commercialized biodiesel satisfies the necessary requirements for its use without damaging engines and mitigating environmental impacts. ASTM D6751-23 sets the maximum concentrations of the combination of metals (e.g., calcium, magnesium, sodium, potassium) in biodiesel at 5 µg/g [19]. Therefore, the removal of these and other trace metals is a fundamental part of the biodiesel production process [15].

Biodiesel purification is mainly carried out using two approaches: wet and dry routes [20]. The typical purification method uses water to remove biodiesel impurities (i.e., wet-based separation) because of its effectiveness and ease of implementation [21,22]. However, its main disadvantage is the utilization of large water volumes, where it has been estimated that 0.2–10 L of wastewater can be generated to purify 1 L of biodiesel [23]. This parameter translates into increments of production costs and time but, more importantly, generates a significant environmental impact because the final wastewater contains high chemical and biological oxygen demand, in addition to other inorganic pollutants depending on catalyst composition, thus creating the necessity of an additional treatment for its proper disposal [21,24]. In contrast, the dry-wash purification process replaces water with a filtration material to remove impurities from biodiesel [25]. This method is faster, reduces wastewater generation, offers operational simplicity and high selectivity if a proper filtration medium is used, and allows for the production of high-quality biodiesel [26]. Membranes, ion-exchange resins, commercial silicate- or sulfate-based adsorbents (e.g., magnesol, calcium silicate, and purolite), silica gel, and activated carbon can be used in the dry washing processes [21,22]. However, the cost of these materials can be a potential drawback, in addition to the properties and synthesis conditions of their available commercial options are not specifically tailored for biodiesel purification, which should imply the application of additional processes to achieve the separation goal [24,25,27].

Adsorbents prepared from residual biomass from agricultural, industrial, and urban sources are an alternative to minimize the cost of biodiesel purification. These adsorbents can also be regenerated and reused, contributing to the sustainability of the separation process [15,25,28]. Spent coffee grounds (CGs) can be used as feedstock to prepare adsorbents for purification tasks in liquids and gases [29]. CGs are wastes generated by approximately 650 kg of every ton of processed coffee. This byproduct is typically discarded in landfills or incinerated, thereby generating environmental pollution [30]. CGs are primarily composed of polysaccharides such as cellulose and hemicellulose (~50% of their composition) followed by lignin. They also contain oils, phenolic compounds, minerals, and tannins [31]. Previous studies have indicated that CGs can be precursors for obtaining activated carbon owing to their high content of carbon-rich components [32,33]. According to the literature review performed for the present study, activated carbons from CGs residues have not been reported for biodiesel purification, particularly to remove metal impurities.

This manuscript reports the tailoring of the adsorption properties of CGs activated carbon to depollute biodiesel containing copper (Cu^2+^). This metal was selected as a model impurity for the biodiesel obtained from heterogeneous catalytic processes. Herein, it is convenient to remark that the development of circular-economy-based processes to obtain renewable energy has promoted the utilization of different wastes and residues containing metallic species as low-cost catalysts for biodiesel production [34]. These catalysts also include materials composed of copper [35], and several studies have indicated that copper-based catalysts prepared from waste conversion and recycling offer additional advantages to obtain biofuels [36]. It is noteworthy that the current legislation does not cover the presence of Cu^2+^ or other metallic elements in biodiesel that can be derived from the application of non-traditional catalysts. Therefore, it is important to obtain novel adsorbents that can handle the pollution generated by emerging catalytic materials. This manuscript reports the assessment of a set of preparation conditions to tailor CGs activated carbons properties to enhance Cu^2+^ adsorption. The best CGs-based adsorbent was tested in both batch and dynamic operation modes and its comparison with respect to a commercial bone char was also performed. The results reported in this manuscript will contribute to achieving the goal of obtaining high-quality, environmentally sustainable biodiesel.

## 2. Results and Discussion

### 2.1. Identification of the Best Synthesis Route for CGs Activated Carbon

Cu^2+^ adsorption capacities of CGs activated carbon samples obtained from different synthesis routes are listed in Table 1. Experimental q_Cu_ values of activated carbon samples ranged from 12.7 mg/g (route No. 3) to 17.8 mg/g (route No. 5). As reference, the Cu^2+^ adsorption capacity of raw CGs biomass was 1.8 mg/g implying that activated carbons showed a significant increment in their Cu^2+^ removal performance that ranged from 609 up to 894%. These results confirmed that the tested preparation conditions favored the formation of surface properties of CGs adsorbents to separate Cu^2+^ ions from biodiesel [37,38].

**Figure 1:**
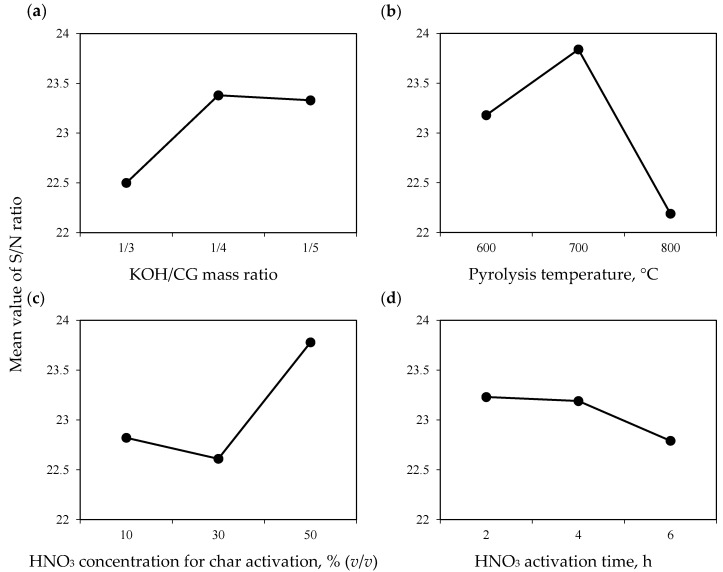
S/N ratio results for the preparation conditions of CGs activated carbon to adsorb Cu^2+^ from biodiesel. (**a**) KOH/CG mass ratio; (**b**) Pyrolysis temperature; (**c**) HNO_3_ concentration for char activation; (**d**) HNO_3_ activation time.

S/N ratio analysis of Taguchi experimental is shown in Figure 1, while ANOVA results are reported in Table 2. It was found that increments on the KOH/CGs mass ratio and HNO_3_ concentration for char activation favored the surface properties of adsorbents to remove Cu^2+^ from the biofuel, while the contrary trend was observed for the CGs pyrolysis temperature and HNO_3_ activation time. The pyrolysis temperature to produce the char was the variable with the statistically greatest effect on Cu^2+^ adsorption capacities of CGs activated carbons followed by HNO_3_ concentration utilized in the char activation, KOH/CG mass ratio, and HNO_3_ activation time.

The pyrolysis temperature that favored the best Cu^2+^ removal performance of CGs adsorbents was 700 °C. It appeared that the degradation of char surface chemistry occurred at >700 °C, leading to the loss of functional groups associated with the metal adsorption capacity [39].

The highest HNO_3_ concentration that was tested for the char surface activation in the different synthesis routes favored the maximization of S/N ratio (i.e., Cu^2+^ adsorption properties). This result was attributed to the formation of a greater number of oxygenated functionalities on the adsorbent structure [40]. 

This point will be discussed in detail using the characterization analyses described below. For the case of KOH/CGs mass ratio, the results indicated that there was no significant difference on the adsorption properties of activated carbon samples obtained with 1:5 and 1:4 ratios. This trend was attributed to the fact that a high amount of KOH can degrade the biomass feedstock used to obtain the activated carbon, affecting its final adsorption properties [41]. Consequently, 1:4 ratio was the best value to minimize the use of KOH for the activated carbon preparation without compromising its Cu^2+^ removal performance. Overall, the time utilized for the char activation with HNO_3_ slightly affected the surface properties of CGs activated carbons to separate Cu^2+^ from biodiesel being the best values between 2 and 4 h. However, 2 h for acid activation was useful to optimize the adsorbent synthesis route, also reducing energy consumption. Note that a prolonged acid activation time of CGs char (e.g., >4 h) affected the adsorbent surface chemistry due to the possible structural degradation [42].

In summary, this statistical analysis allowed for the conclusion that the main variables that participate in the formation of functional groups on CGs activated carbons for enhancing Cu^2+^ adsorption capacity were the pyrolysis temperature to obtain the char and its corresponding acid activation. These results agreed with previous studies on carbon-based adsorbent preparation [43]. The maximization of S/N ratio indicated that the synthesis route No. 5 was the best to tailor the surface properties of CGs activated carbon to remove Cu^2+^ from biodiesel. This route involved the next preparation conditions: 1:4 of KOH/CGs mass ratio, biomass pyrolysis at 700 °C, 50% (*v*/*v*) HNO_3_ concentration and 2 h to activate the CGs char.

### 2.2. Surface Chemistry and Composition of CGs and Their Activated Carbons

ICP analysis indicated that the main elements contained in raw CGs were Ca, Mg, K, and Na, see Table 3. These results were consistent with the information reported in the literature for this residual biomass [44]. A significant change was observed in the composition of CGs activated carbons particularly in K content, which was attributed to the mixing of CGs biomass with KOH before its pyrolysis. Note that KOH and HNO_3_ may also contribute to the presence of some impurities (e.g., other metallic species) in the adsorbents, while the remaining elements were found in traces and their origin was mainly associated with the raw materials used in the activated carbon preparation.

**Figure 2:**
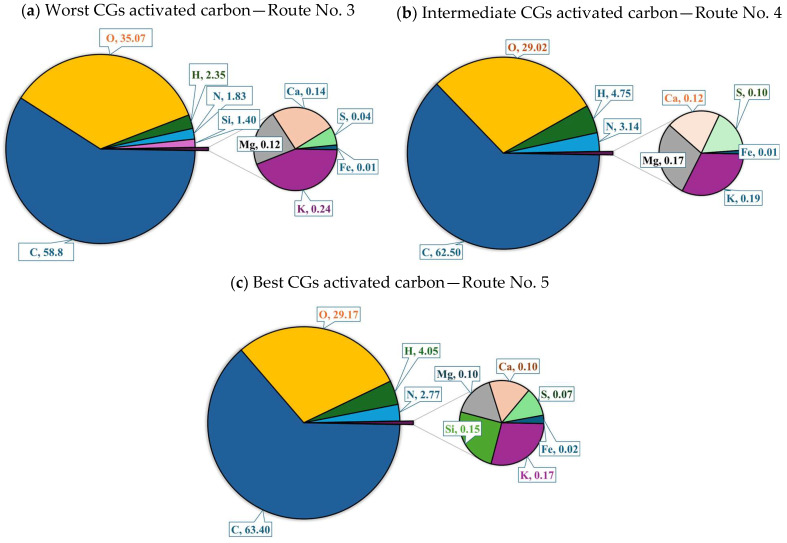
Elemental composition of CGs activated carbons used to adsorb Cu^2+^ from biodiesel.

The results of organic elemental analyses for CGs activated carbon samples from routes No. 5 (the best adsorbent), No. 4 (intermediate performance), No. 3 (the worst adsorbent) are reported in Figure 2. These samples were characterized by their high content of carbon (58.8–63.4 wt%) and oxygen (29.2–35.1 wt%), and low content of hydrogen (2.4–4.8 wt%) and nitrogen (1.8–3.1 wt%). EDX and XPS analyses also confirmed the elemental composition of tested adsorbents including C, O, H, and N contents, see Tables S1 and S2 in Supporting Information.

C content increased in the adsorbents compared to the precursor material, and it decreased when CGs was treated with KOH and pyrolyzed. Note that EDX also confirmed that the origin of trace elements was the raw biomass used in the activated carbon preparation.

**Figure 3:**
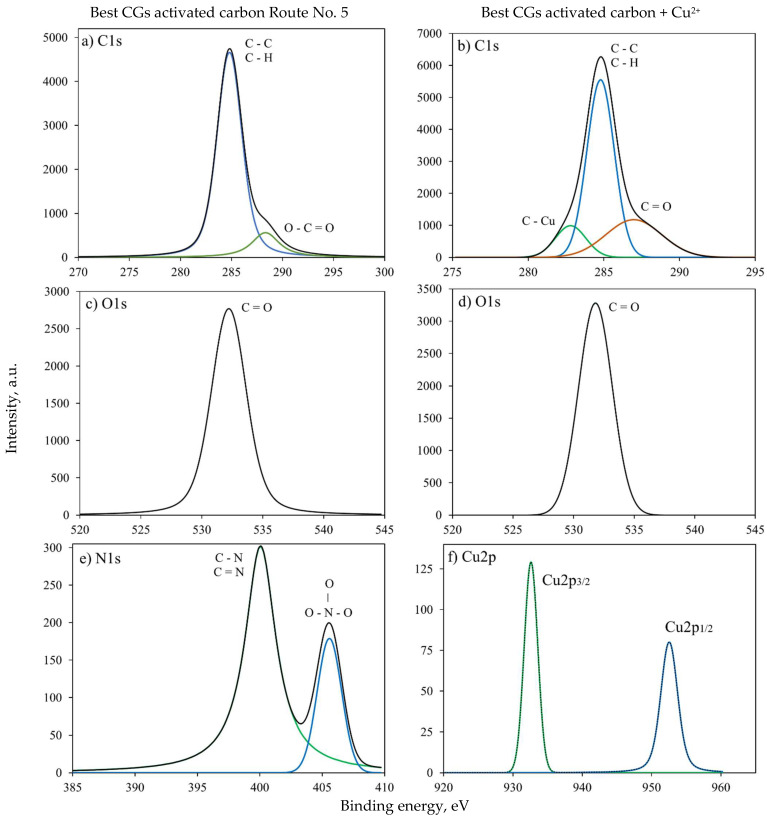
X-ray photoelectron spectroscopy analysis of the best CGs activated carbon before and after Cu^2+^ adsorption. (**a**,**b**) C1s, (**c**,**d**) O1s, (**e**) N1s, and (**f**) Cu2_p_.

For illustration, Figure 3 and Table S3 show the results of XPS spectra deconvolution and the calculated areas for the best CGs activated carbon before and after Cu^2+^ adsorption, respectively. Specifically, the C1s orbital exhibited peaks with a maximum near to 284.8 eV, which was associated with various oxidized carbon forms. The peak at 284.8 eV corresponded to C-C bonds and aromatic bonds of the basal planes of carbon, aliphatic hydrocarbons, and C-H bonds [45], while the peaks centered at 287.0, and 288.7 eV corresponded to oxidized C. The first peak was associated with C bonded to O by a double bond (C=O), and the second to C bonded to O by both single and double bonds (O-C=O).

The binding energy of 282.8 eV identified for the CGs activated carbon after Cu^2+^ adsorption was related to the carbon-metal bond (i.e., copper). For the case of N1s orbital, the peaks located at 400.1 and 405.6 eV were attributed to the oxidized nitrogen in the CGs activated carbon. The peak at 400.1 eV was assigned to nitrogen in an intermediate oxidation state (C-N, C=N), and the peak at 405.6 eV indicated the presence of nitrate (NO_3_) [46]. Herein, it is convenient to recall that the interpretation of the XPS spectra of O1s in organic materials is sometimes complex. The binding energy at 532.1 eV was assigned to the double bond of organic carbon with oxygen.

Note that the Cu2_p_ orbital displayed two peaks at 932.6 and 952.5 eV, corresponding to Cu2_p3/2_ and Cu2_p1/2_, respectively [45]. Therefore, XPS results of CGs activated carbon after adsorption in biodiesel confirmed the presence of Cu^2+^ on the material surface.

**Figure 4:**
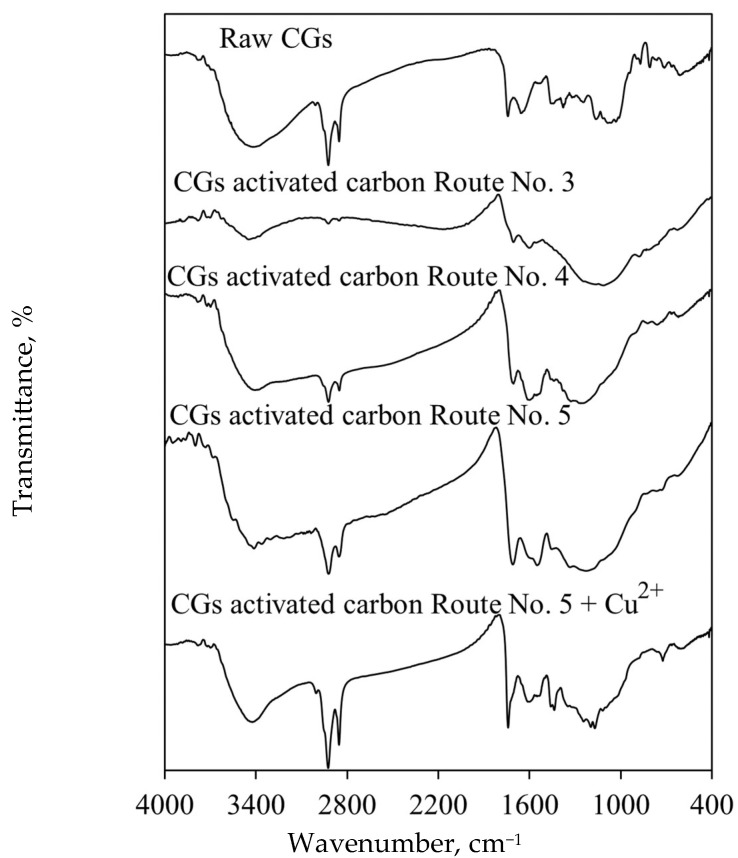
FTIR spectra of raw CGs and CGs activated carbon samples used in Cu^2+^ adsorption from biodiesel.

Figure 4 shows FTIR spectra of raw CGs and selected samples of activated carbons before and after Cu^+2^ adsorption. In the CGs spectrum, an absorption band at 3412 cm^−1^ was identified corresponding to -OH groups from alcohols, phenols, cellulose, hemicellulose, and lignin. The absorption band located at 3600 cm^−1^ overlapped with the previous band and was associated with -NH groups of amino acids from caffeine and proteins in the CGs biomass [47,48,49,50]. The absorption bands identified at 2918, 2850, and 1439 cm^−1^ were attributed to the stretching of C-H groups associated with aliphatic chains, possibly from caffeine, lipids, cellulose, and hemicellulose contained in CGs [47,48]. 

C=O groups from esters were identified with the absorption band at 1739 cm^−1^, whereas the absorption band at 1375 cm^−1^ was related to the glycosidic bonds in cellulose and hemicellulose [51]. Various sharp absorption bands were observed in the region of 900–500 cm^−1^, which were attributed to the presence of polysaccharides (e.g., mannose, glucose, arabinose, galactose) [52]. The absorption band at 1647 cm^−1^ was related to the caffeine residues from CGs and corresponded to the C=C and C=O groups present in aromatic structures [52].

Regarding the surface chemistry of CGs activated carbons, FTIR spectrum of the adsorbent with the lowest Cu^2+^ adsorption capacity contained absorption bands with low intensity for the oxygenated functional groups. It is convenient to remark that this type of activated carbon functionalities is recognized as the main active sites for the adsorption of metal ions [53,54].

Conversely, an increase in the intensity of these bands was observed for the adsorbent with better Cu^2+^ adsorption properties. The absorption band at 1700 cm^−1^ corresponding to the C=O bond of carboxylic groups was recorded, which was associated with the char acid activation. The vibrations of C-O bonds were attributed to the absorption band at 1450–1000 cm^−1^ and corresponded to the presence of ethers, esters, alcohols, and phenols [47,50].

The absorption band at 1360 cm^−1^ corresponding to the N-H group overlapped with the mentioned region [49]. These results confirmed that the pyrolysis and HNO_3_ activation generated functional groups on CGs surface to improve Cu^2+^ adsorption properties [37,38]. Overall, a change was observed in the content of oxygenated functional groups on the surface of different activated carbon samples. These functionalities are strong acceptors of cations, rendering the surface predominantly acidic, primarily due to the presence of carboxyl and hydroxyl groups [37,38].

FTIR spectrum of the tailored CGs activated carbon (i.e., highest adsorption capacity) contained absorption bands of greater amplitude and intensity than that observed for other adsorbent samples, particularly, at 1720–1050 cm^−1^. This result was evidence of a higher content of carboxylic groups [55], which were correlated with the Cu^2+^ adsorption properties of tested adsorbents. FTIR analyses of adsorbent samples after Cu^2+^ adsorption from biodiesel showed a significant decrease in the absorption band intensity at 3412 cm^−1^ corresponding to -OH group. Additionally, the absorption band at 1700 cm^−1^ shifted to 1744 cm^−1^, suggesting an interaction between Cu^2+^ ions and carboxyl groups of CGs activated carbon surface [56,57]. There was also a decrease in the absorption band intensity in the region from 1400 to 1194 cm^−1^ [58], while the absorption band located at 721 cm^−1^ was attributed to the Cu-O bond [59]. Note that this absorption band appeared only after Cu^2+^ adsorption from biodiesel using tested adsorbents.

**Figure 5:**
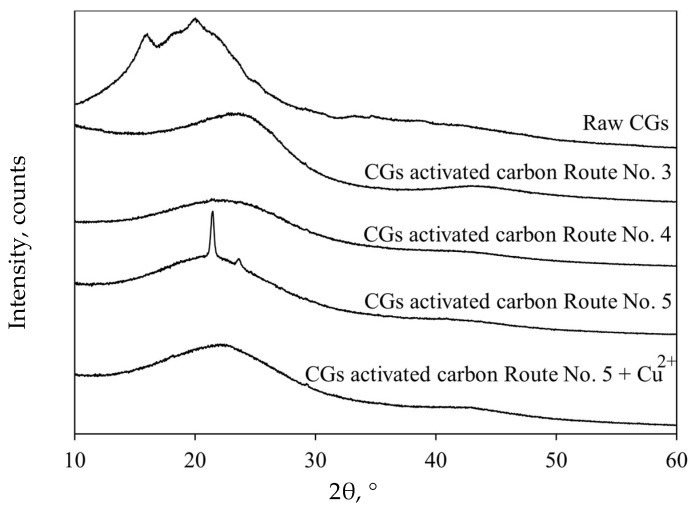
X-ray diffraction results of raw CGs and CGs activated carbon samples used in Cu^2+^ adsorption from biodiesel.

Figure 5 shows the X-ray diffractograms for both CGs biomass and selected adsorbent samples. Particularly, the characteristic diffraction peaks associated with the crystallinity of cellulose and hemicellulose [60] were observed in the diffraction pattern of raw CGs at 15 and 22° 2Ɵ, respectively. The diffractograms of CGs activated carbons exhibited the typical structure of carbonaceous materials with peaks located at 24 and 42° 2Ɵ, the latter with lower intensity and associated with the amorphous structure of graphite [61]. A diffraction pattern with slightly more pronounced peaks was observed for the CGs activated carbon with the worst performance for Cu^2+^ adsorption from biodiesel. This result showed a higher adsorbent crystallinity due to surface degradation caused mainly by the pyrolysis temperature. On the other hand, the presence of two peaks at 21.5 and 23.7° 2Ɵ was observed in the diffraction pattern of the best CGs activated carbon. These peaks were associated with the calcium contained in the precursor [45,62]. After Cu^2+^ adsorption, an increase in the diffractogram intensity of activated carbons was observed, indicating a slight change in their crystallinity.

**Figure 6:**
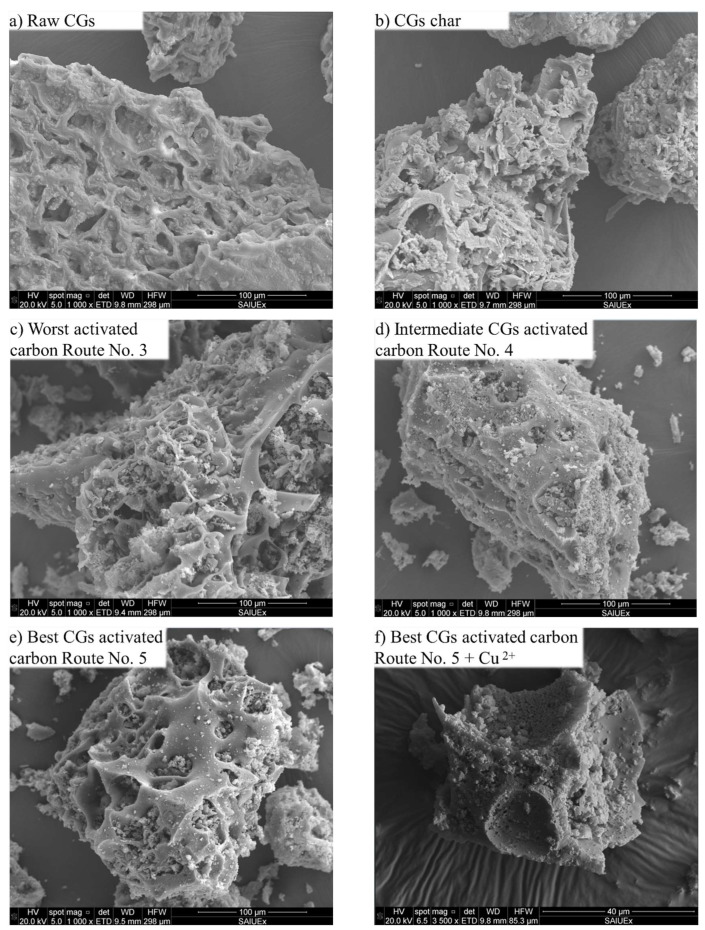
SEM images of raw CGs, char and activated carbons used in Cu^2+^ adsorption from biodiesel.

SEM-based morphology analysis of CGs and its char and activated carbon forms is reported in Figure 6. For raw CGs and char, particles of varying sizes were observed where large ones predominated. For all adsorbents, the pore mouths, especially the larger ones, were filled with particulate matter, which was more abundant in the worst CGs activated carbon sample. It is worth noting that this particulate matter in the pores was not observed in the CGs char. The results indicated that the impact of HNO_3_ treatment on surface morphology of the adsorbents was different. The micrographs of the char and activated carbons confirmed their surface changes. For example, an irregular eroded surface was visible for the char sample because of pyrolysis and KOH treatment, while a highly eroded and porous surface was observed for tested activated carbon samples. In comparison to char, the activated carbons showed unclogged cavities and the exposure of inner layers. This morphology was more evident for the worst activated carbon (see Figure 6c), where particulate matter was observed. It is noteworthy that this activated carbon was prepared under the most extreme conditions of pyrolysis temperature, HNO_3_ concentration and acidic activation time.

**Figure 7:**
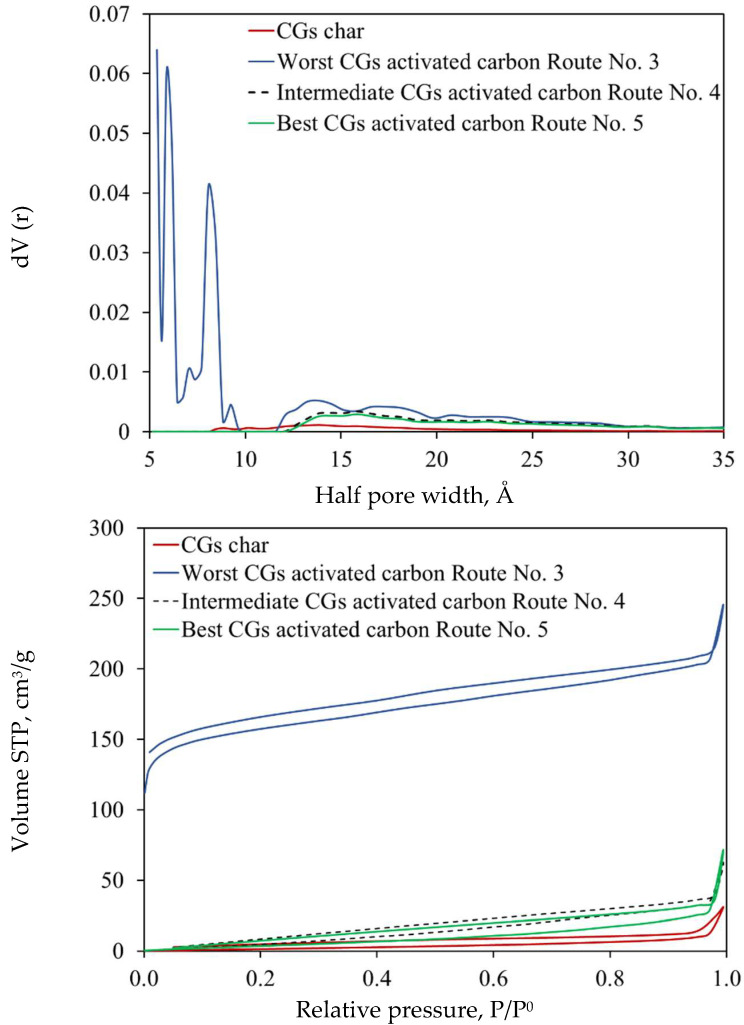
Nitrogen isotherms and pore distribution of CGs activated carbons used in Cu^2+^ adsorption from biodiesel.

**Figure 8:**
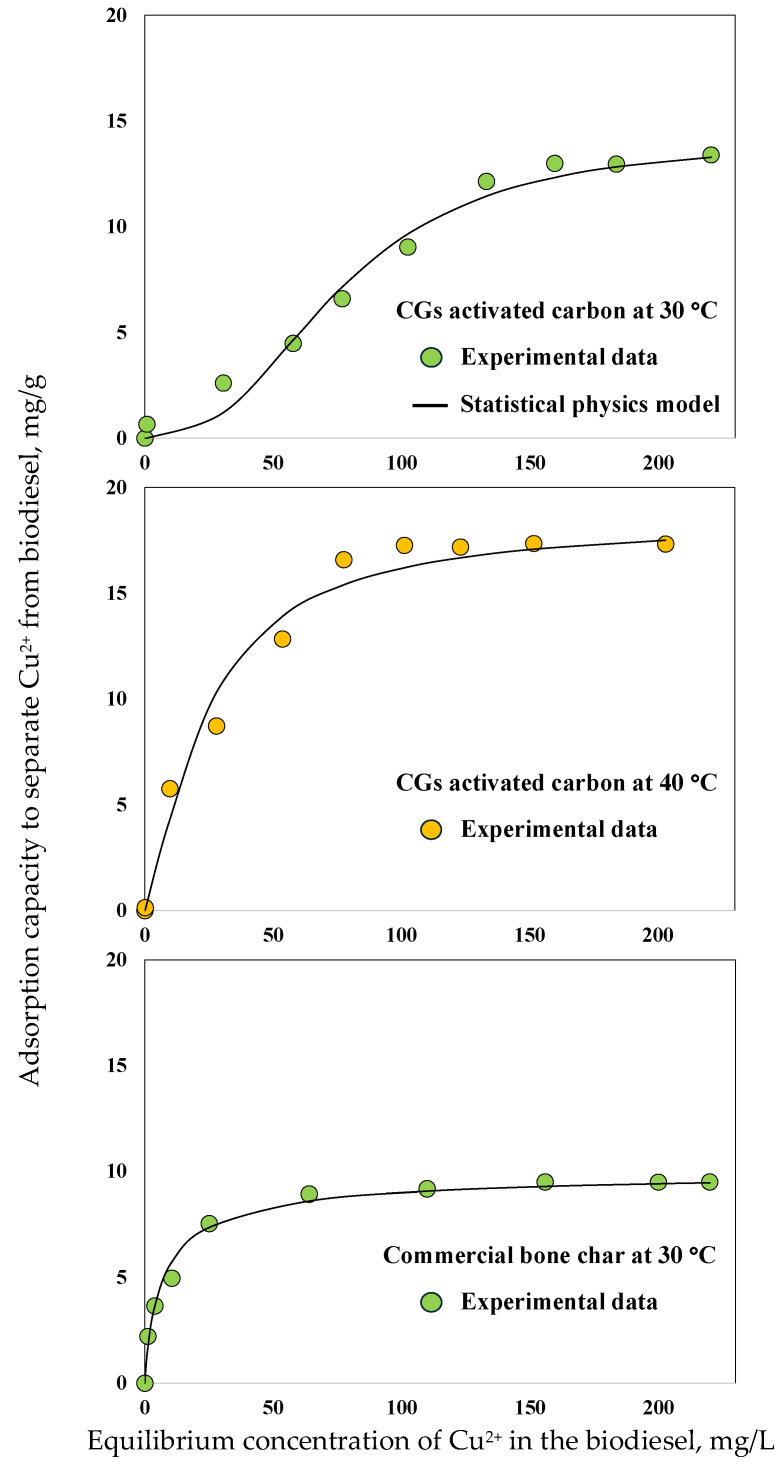
Adsorption isotherms for Cu^2+^ separation from biodiesel using the best CGs activated carbon and commercial bone char.

Finally, N_2_ adsorption isotherms indicated that CGs activated carbons exhibited no development of microporosity and a small degree of mesoporosity, except for the worst adsorbent, see Figure 7. This worst adsorbent was the only sample that presented a significant volume of both micro- and mesopores, with the latter being slightly more prominent. The summary of adsorbent textural parameters is given in Table 4. Specific surface area of CGs activated carbons was up to 495 m^2^/g. The analysis of pore size distribution (Figure 8) demonstrated that CGs activated carbon only presented narrow pores < 30 Å. The char and best activated carbon mainly exhibited pore sizes between 12 and 20 Å, while the worst adsorbent showed a broader pore size range: 5–10 and 12–25 Å. This information was consistent with the SEM-based morphology results.

### 2.3. Batch and Continuous Experiments and Modeling of Cu^2+^ Adsorption from Biodiesel

**Figure 9:**
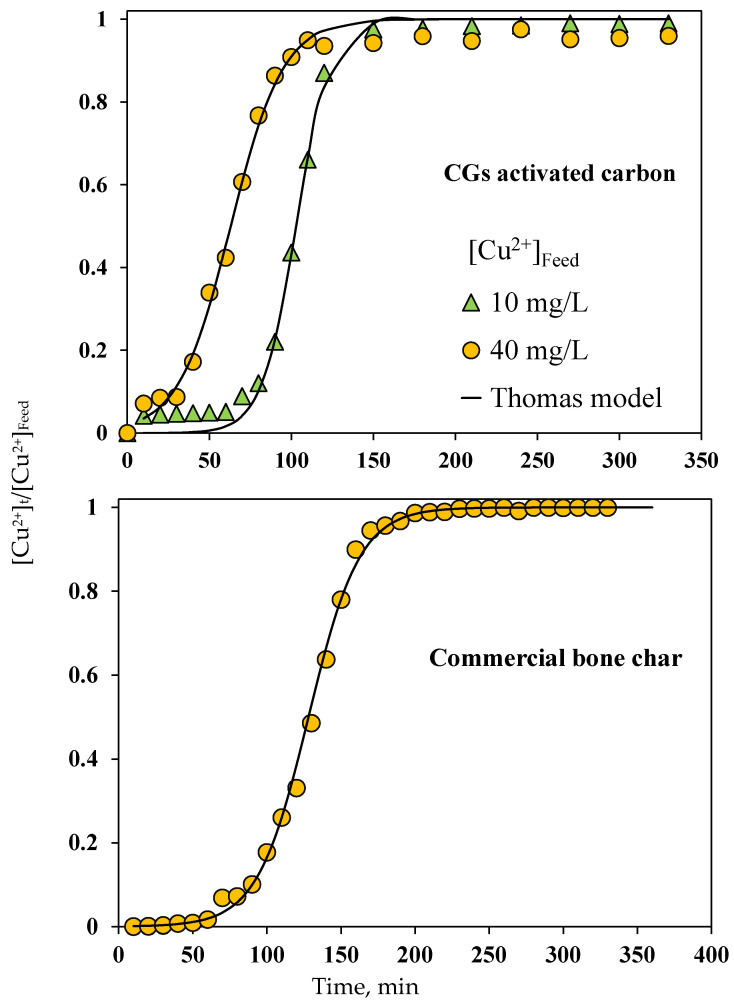
Breakthrough curves for Cu^2+^ adsorption from biodiesel using columns packed with the best CGs activated carbon and commercial bone char.

The best tailored adsorbent was studied in batch and dynamic configurations to calculate the main thermodynamic and packed-bed column parameters, and the results are reported in Figure 8 and Figure 9. In particular, the isotherms for Cu^2+^ adsorption from biodiesel using the best CGs activated carbon operating at 30 and 40 °C are reported in Figure 8. The *q_Cu,Iso_* was 13.4 and 17.3 mg/g at 30 and 40 °C, respectively. The separation of Cu^2+^ from biodiesel using this adsorbent was endothermic where the adsorbent removal capacity improved in 29% with the temperature increment. This endothermic behavior was attributed to the fact that biodiesel heating granted Cu^2+^ ions a higher freedom of movement, facilitating their mass transfer in the internal pore structure of the adsorbent to interact with its active sites [63].

Additionally, the biodiesel viscosity decreased with the temperature increase, which reduced the boundary layer surrounding the adsorbent and, thereby, the mass transfer for Cu^2+^ adsorption was enhanced [30,64,65]. The ΔH = 21 kJ/mol was estimated for this endothermic separation process [57,66]. This adsorption enthalpy indicated the presence of physical adsorption forces and agreed with the characterization results of CGs activated carbon. Note a previous study reported ΔH = 10 kJ/mol for Cu^2+^ adsorption from water using raw CGs biomass [67].

Table 5 presents the fitting results of equilibrium data using the Langmuir, Freundlich, and Sips models. The Sips model showed the best correlation with modeling errors of 4.8 and 4.3% and R^2^ = 0.96 and 0.94 for the isotherms at 30 and 40 °C, respectively. This model is considered as a combination of Freundlich and Langmuir isotherms and has been proposed to describe adsorbents with heterogeneous surfaces as activated carbons [68]. Note that the heterogeneous surface of CGs activated carbon was associated with the presence of various oxygenated functional groups [55]. However, the carboxylic groups acted as the main anchoring sites for Cu^2+^ ions from biodiesel as confirmed by characterization results. Calculated *n_Sips_* values were close to 1 suggesting a Langmuir-type isotherm via monolayer adsorption [68,69]. Based on these results, a statistical physics model was utilized to complement the Cu^2+^ adsorption mechanism analysis. This model assumed that Cu^2+^ adsorption from biodiesel on the best CGs activated carbon implied the formation of a monolayer with one energy (Δ*e*, kJ/mol) for the cation–carboxylic group interaction

(1) $$q_{Cu}=\frac{n_{Cu}\cdot D_{CGs}}{1+{\left(\frac{{\left[{Cu}^{2+}\right]}_{1/2}}{{\left[{Cu}^{2+}\right]}_{e}}\right)}^{n_{Cu}}}$$

where [Cu^2+^]_1/2_ is the half-saturation Cu^2+^ concentration, [Cu^2+^]_e_ is the equilibrium Cu^2+^ concentration, *n_Cu_* represents the number of Cu^2+^ ions adsorbed per carboxylic group on CGs activated carbon surface, and *D_CGs_* is the estimated concentration of carboxylic groups that participated in the Cu^2+^ adsorption from biodiesel. Δ*e* was calculated using the next expression

(2) $$\Delta e=RTln\left(\frac{S_{Cu}}{{\left[{Cu}^{2+}\right]}_{1/2}}\right)$$

where *T* is the adsorption temperature in Kelvin, *R* is the universal gas constant, and *S_Cu_* is the Cu^2+^ solubility under the tested operating conditions.

The isotherm data correlation with the statistical physics model was performed in mmol-based units via a non-linear regression. Calculated *n_Cu_* values were 2.6 and 1.35 at 30 and 40 °C. Therefore, a multi-cationic mechanism may occur to separate Cu^2+^ from biodiesel using CGs activated carbon. The calculated concentration of carboxylic groups participating in the metal separation from biofuel was 0.09 and 0.22 mmol/g at 30 and 40 °C. Therefore, an increment of CGs activated carbon functionalities was observed due to the fluid temperature change, indicating that the mass transfer was enhanced by the thermal agitation allowing for more carboxylic groups from the internal adsorbent pore structure to be involved in Cu^2+^ adsorption. The saturation condition of carboxylic groups of this CGs activated carbon corresponded to theoretical Cu^2+^ adsorption capacities of 14.1 and 18.5 mg/g at 30 and 40 °C, respectively.

Calculated Δ*e* values were 6–8 kJ/mol confirming a physical-type interaction to remove Cu^2+^ cations from biodiesel via the activated carbon carboxylic groups. Note that biodiesel has a pH ~6, while the pHpzc of the adsorbent is 4.57 and the pKa of carboxylic groups is ~5. Consequently, the adsorbent surface was negatively charged and these functionalities were deprotonated at tested operating conditions causing that the electrostatic interactions prevailed for Cu^2+^ separation during biofuel purification

≡CGs-COO^−^ + *n*_Cu_Cu^2+^ → ≡CGs-COO^−^ ⋅⋅⋅(*n*_Cu_Cu^2+^) (3)

It is convenient to recall that the statistical physics calculations indicated that up to *n*_Cu_ = 3 cations can interact per each carboxylic active site from the adsorbent surface.

Experimental breakthrough curves for Cu^2+^ purification of biodiesel using the tailored CGs adsorbent are reported in Figure 9 where the characteristic “S” shape was observed under tested operating conditions. Table 6 lists the breakthrough curve parameters for the best CGs activated carbon. Calculated *q_Cu,Bed_* values were 2.1 and 5.1 mg/g for feed concentrations of 10 and 40 mg/L, respectively. On the other hand, t_b_ was 72.9 and 31.5 min and MTZ was 3.6 and 5.9 cm at [Cu^2+^]_Feed_ = 10 and 40 mg/L, respectively, while *t_e_* decreased from 150 to 100 min. These parameters proved that the feed column concentration was a determining factor affecting the biodiesel purification. Overall, the breakthrough point time was faster at higher [Cu^2+^]_Feed_ because the CGs activated carbon saturated more quickly [70]. The increase in Cu^2+^ concentration in the biodiesel generated an increment of MTZ, ΔT and r_F_ values because of the impact of concentration gradient on mass transfer in the packed-bed column via the reduction in Cu^2+^ diffusion resistance [69,71,72]. The degree of adsorbent utilization in the packed-bed column also increased from 14 to 38% with [Cu^2+^]_Feed_ change. As expected, q_Cu,Bed_ < q_Cu,Iso_ highlighting the impact of axial dispersion on the purification performance of fixed-bed columns packed with CGs activated carbon. Calculated parameters of the Thomas model are also reported in Table 6. This breakthrough model satisfactorily fitted Cu^2+^ experimental data with R^2^ = 0.99 and the maximum modeling error of 2.23%. The adsorption capacities obtained by the Thomas model were similar to those obtained via the numerical integration of experimental breakthrough data.

Herein, it is convenient to remark that there are a lack of studies reporting the removal of heavy metals from biodiesel because the common separation targets are glycerin, alcohol, soap, and water [73,74]. For example, Squissato et al. [15] analyzed Cu^2+^ adsorption from biodiesel using 1 g of bleached eucalyptus pulp packed in a column with dimensions of L = 10 cm and D = 1.3 cm. This material showed a low adsorption capacity (i.e., <0.1 mg/g), which was significantly outperformed by that of the CGs adsorbent prepared in this study.

On the other hand, the results of Cu^2+^ separation using the commercial bone char are reported in Figure 8 and Figure 9. This adsorbent showed q_Cu,Iso_ = 9.5 mg/g at 30 °C and q_Cu,Bed_ = 1.2 mg/g at [Cu^2+^]_Feed_ = 10 mg/L, while the calculated breakthrough parameters for bone char packed-bed column are summarized in Table 6. All of these values confirmed that the tailored CGs adsorbent outperformed the commercial bone char to depollute biodiesel containing Cu^2+^ ions. As indicated, bone char is a very effective adsorbent to remove Cu_2+_ from water with adsorption capacities up to 45.8 and 127.7 mg/g for batch and continuous experiments, respectively [75,76]. However, its performance for the separation of Cu^2+^ cations from biodiesel significantly reduced due to the mass transfer resistances generated by the biodiesel properties. This comparison highlights the importance of tailoring the surface chemistry of adsorbents to be used in the purification of biofuels polluted by the application of both homogeneous and heterogeneous catalysts.

## 3. Materials and Methods

### 3.1. Preparation of Biodiesel Polluted with Cu^2+^

Biodiesel was synthesized via the transesterification of commercial safflower oil with methanol at a molar ratio of 1:15, using 1 wt% KOH catalyst at 60 °C for 5 h under constant stirring. The biodiesel was recovered by decantation and placed in an oven at 100 °C to evaporate any unreacted alcohol. Subsequently, the biodiesel was washed twice with 1% (*v*/*v*) HNO_3_ solution and 20% (*v*/*v*) water to remove the catalyst [77,78]. The sample was then dried at 100 °C for 12 h. Cu(NO_3_)_2_ was added to the biodiesel for obtaining the set of initial concentrations used in the Cu^2+^ adsorption studies.

### 3.2. Tailoring of Surface Properties of CGs Activated Carbon to Remove Cu^2+^ from Biodiesel

CGs wastes were used as the precursor biomass to prepare activated carbon samples with different surface properties to remove Cu^2+^. CGs particles were homogenized by sieving to obtain an average size of 0.5 mm. Biomass residues were washed with deionized water at 80 °C until a constant pH was reached in the residual liquid, and they were then dried at 100 °C for 12 h. Oil extraction from the CGs was performed using hexane under constant stirring at 50 °C for 30 min [79], followed by the particle drying at 100 °C for 24 h. This clean residual biomass was utilized in the synthesis of all adsorbents.

**Figure 10:**
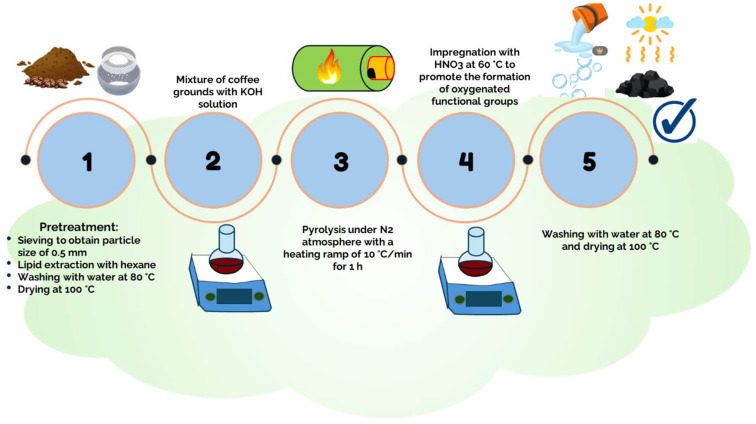
Procedure used to obtain the activated carbons from CGs with tailored properties to remove Cu^2+^ from biodiesel.

A set of activated carbons was obtained from CGs using different preparation conditions. These adsorbents were synthesized by mixing CGs with a KOH solution, and this modified biomass was pyrolyzed under N_2_ atmosphere with a heating ramp of 10 °C/min for 1 h to obtain a char. This char was then activated with a HNO_3_ solution under constant stirring at 60 °C for a specific time to promote the formation of oxygenated functional groups on the adsorbent surface. Finally, CGs activated carbons were washed with deionized water until the washing liquid reached a constant pH, and the samples were dried in an oven at 100 °C for 24 h. Figure 10 provides a flowchart of the main steps involved in the CGs activated carbon preparation.

Table 1 shows the Taguchi L_9_ experimental design applied to study the preparation conditions of CGs activated carbon samples. The main variables analyzed for tailoring the Cu^2+^ adsorption properties of CGs activated carbon samples were: KOH/CGs mass ratio, the pyrolysis temperature of KOH-modified CGs biomass to obtain a char, HNO_3_ solution concentration used to activate the char obtained from pyrolysis, and its corresponding HNO_3_ activation time. The tested preparation conditions are reported in Table 1, which were selected by considering the results of other studies on CGs valorization [38].

The response variable of the experimental design was the Cu^2+^ adsorption capacity (*q*_Cu_, mg/g) of CGs activated carbons. The *q*_Cu_ values were determined using batch adsorbers with an initial Cu^2+^ concentration [Cu^2+^]_0_ of 200 mg/L in the biodiesel and 0.02 g/mL of activated carbon dosage at 40 °C for 24 h under constant stirring. Equation (S1) from the Supporting Information was applied to calculate *q*_Cu_. The quantification of Cu^2+^ in the biodiesel was performed via atomic absorption spectrophotometry operated in flame mode, see more details in the Supporting Information. Prior to the metal determination, all biodiesel samples, performed in triplicate, were subjected to acidic digestion with HNO_3_ and H_2_O_2_ to demineralize the organic phase. The statistical analysis of Taguchi experimental design was carried out via the signal-to-noise (S/N) ratio, which was calculated for *q*_Cu_ applying the “larger value, better response” premise [80]

(4) $$\frac{S}{N}=-10Log\left[\frac{1}{n_{rep}}\sum_{i=1}^{n_{rep}}\frac{1}{{q_{Cu}}_{i}^{2}}\right]$$

where S/N ratio was obtained from the replicates *n*_rep_ of each experiment *i*, see Table 1. An analysis of variance (ANOVA) allowed for the identification of synthesis conditions that significantly impacted the adsorption properties of CGs activated carbon to depollute biodiesel containing Cu^2+^. ANOVA results were also applied to select the best synthesis route for tailoring the Cu^2+^ removal performance of CGs activated carbon. Note that additional batch and continuous adsorption experiments were carried out using the best CGs activated carbon, as indicated below.

### 3.3. Batch and Fixed-Bed Adsorption of Cu^2+^ from Biodiesel

Cu^2+^ adsorption isotherms were quantified using the best CGs activated carbon at 30 and 40 °C. These equilibrium tests were performed with [Cu^2+^]_0_ = 10–600 mg/L where CGs activated carbon and biodiesel were mixed at a ratio of 20 g/L for 24 h under constant stirring. Continuous experiments to remove Cu^2+^ from the biofuel were also carried out via a fixed-bed adsorption micro-column with a bed length (*L*) of 7 cm and an internal column diameter (*D*) of 1.1 cm. 2.7 g of CGs activated carbon (*m_Bed_*) were packed and the bed porosity (*ε*) was ~30%. The columns were operated with a biodiesel flow rate (*Q*) of 0.18 L/h and an initial feed Cu^2+^ concentration [Cu^2+^]_Feed_ of 10 and 40 mg/L. Effluent samples were collected every 10 min during the first 2 h and every 30 min thereafter until the adsorption process was complete to treat 2 L of Cu^2+^-polluted biodiesel. These results were used to obtain the corresponding breakthrough curves. All fixed-bed experiments were performed in triplicate.

The equilibrium data were correlated with the Langmuir [81], Freundlich [82], and Sips [83] isotherm models. The Supporting Information File provides the description of these models. The results from these adsorption models were complemented with statistical physics calculations [84] to improve the discussion of the adsorption mechanism for biodiesel purification. On the other hand, the adsorption enthalpy (Δ*H*, kJ/mol) was estimated from the Cu^2+^ adsorption isotherms via the van ’t Hoff method using the procedure proposed by Tran et al. [85].

For the case of Cu^2+^ adsorption breakthrough curves, the bed Cu^2+^ adsorption capacity (*q_Cu,Bed_*), mass transfer zone (*MTZ*), breakthrough time (*t_b_*), general adsorption zone (Δ*T*), retardation factor (*r_F_*) and degree of CGs activated carbon utilization (*F_q_*) were calculated as follows [86]:

(5) $$q_{Cu,Bed}=\int_{t=0}^{t}\left({\left[{Cu}^{2+}\right]}_{Feed}-{\left[{Cu}^{2+}\right]}_{t}\right)\frac{Q}{m_{Bed}}\;dt$$

(6) $$MTZ=L\left(\frac{t_{e}-t_{b}}{t_{e}}\right)$$

(7) $$\Delta T=t_{e}-t_{b}$$

(8) $$r_{F}=\frac{V_{50\%}}{AL\varepsilon }$$

(9) $$F_{q}=\frac{q_{Cu,Bed}}{q_{Cu,Iso}}$$

where *t_e_* is the saturation time for the packed bed of CGs activated carbon (min), *t_b_* is the breakthrough time (min), [Cu^2+^]_t_ is the effluent Cu^2+^ concentration at time *t*, *V*_50%_ is the treated biodiesel volume (mL) when the Cu^2+^ concentration reaches [Cu^2+^]_t_/[Cu^2+^]_Feed_ = 0.5, *A* is the cross-sectional adsorption column area (cm^2^), and *q_Cu,Iso_* is the maximum Cu^2+^ adsorption capacity obtained from the experimental isotherm. Note that *t_b_* was defined as [Cu^2+^]_t_/[Cu^2+^]_Feed_ = 0.1 for the data analysis. Numerical integration via the trapezoidal rule was used to solve Equation (2). The Thomas equation [87] was applied to fit the experimental breakthrough curves for the Cu^2+^ adsorption from biodiesel. Data fitting of all models for both isotherms and breakthrough curves were performed via non-linear regressions.

Cu^2+^ separation efficacy of the best CGs activated carbon was compared with that of a commercial Brazilian bone char. This commercial adsorbent was produced from the calcination of beef bones under a controlled oxygen atmosphere and its main composition include hydroxyapatite, calcium carbonate and sulfate with a bulk density of 0.65 g/cm^3^ [88]. The adsorbent comparison included both isotherm and fixed-bed experiments under the same operating conditions as reported for CGs activated carbon. Bone char has been recognized as an outstanding adsorbent for removing heavy metals from water [89]. Therefore, this commercial bone char was considered a proper basis to assess the Cu^2+^ adsorption properties of CGs activated carbon in biodiesel.

### 3.4. Surface Chemistry Characterization of CGs Activated Carbon

Samples of CGs activated carbon were characterized to determine their surface chemistry and composition. The main functional groups of these samples were analyzed by Fourier-transform infrared spectroscopy (FTIR) using KBr pellets. X-ray diffraction (XRD) patterns were obtained to establish the degree of crystallinity and phase identification in the CGs activated carbon. Surface characterization of tested samples was completed with X-ray photoelectron spectroscopy (XPS). The inorganic elemental composition of selected samples was obtained via Inductively Coupled Plasma Spectroscopy (ICP), where the adsorbents were predemineralized by acidic digestion with HNO_3_ and H_2_O_2_. C, H, and N contents of the adsorbent samples were quantified via combustion elemental analysis, while wavelength dispersive X-ray fluorescence (WDXRF) was used to complement the adsorbent composition analysis. The oxygen content was calculated from the difference using the composition analysis results. The morphology of CGs activated carbon samples was recorded using the SEM/EDX technique, while their textural parameters were obtained from the data processing of N_2_ adsorption isotherms. The pH at point of zero-charge (pHpzc) of the best adsorbent was determined using the methodology of Faria et al. [90] and Kalavathy et al. [91]. Details of the instruments and conditions used in the sample characterization are provided in the Supporting Information File.

## 4. Conclusions

A tailored activated carbon for purification of biodiesel polluted by copper was obtained from spent coffee grounds. This novel adsorbent was synthesized via biomass pyrolysis and chemical activation using KOH and HNO_3_ where the best preparation route was identified via a Taguchi experimental design. The statistical analysis of activated carbon preparation conditions indicated that the biomass pyrolysis temperature and HNO_3_ concentration for chemical activation were the main variables to improve Cu^2+^ adsorption properties via the formation of more carboxylic functional groups on the adsorbent surface. This tailored adsorbent showed an endothermic performance to separate Cu^2+^ from biodiesel with a multi-ionic adsorption mechanism where the deprotonated carboxylic groups were the main active sites. Packed-bed adsorption studies indicated a degree of adsorbent utilization of 14–38% depending on the column feed concentration where axial dispersion affected the mass transfer during Cu^2+^ separation in the adsorption columns. The best activated carbon prepared from spent coffee grounds outperformed a commercial bone char in the separation of copper from biodiesel in both batch adsorbers and packed-bed columns. This novel adsorbent can be utilized as a better purification strategy to reduce the concentration of metal impurities in biodiesel obtained from homogeneous and heterogeneous catalytic routes.

## Figures and Tables

**Table 1:** Taguchi L9 experimental design for the preparation of CGs activated carbons to adsorb Cu^2+^ from biodiesel.

Synthesis Route	KOH/CGs Mass Ratio	CGs Pyrolysis Temperature, °C	HNO_3_ Concentration for Char Activation, % (*v*/*v*)	HNO_3_ Activation Time, h	q_Cu_, mg/g
1	1/3	600	10	2	13.4
2	1/3	700	30	4	14.0
3	1/3	800	50	6	12.7
4	1/4	600	30	6	13.7
5	1/4	700	50	2	17.8
6	1/4	800	10	4	13.1
7	1/5	600	50	4	16.3
8	1/5	700	10	6	15.1
9	1/5	800	30	2	12.8

**Table 2:** ANOVA results for the Taguchi L_9_ experimental design used in the preparation of CGs activated carbons to adsorb Cu^2+^ from biodiesel.

Synthesis Variable	Value	Mean S/N Ratio	Variance
KOH/CG mass ratio	1/3	22.5	1.45
	1/4	23.4	
	1/5	23.3	
Pyrolysis temperature, °C	600	23.2	4.09
	700	23.8	
	800	22.2	
HNO_3_ concentration for char activation, % (*v*/*v*)	10	22.82	2.32
	30	22.61	
	50	23.78	
HNO_3_ activation time, h	2	23.23	0.35
	4	23.19	
	6	22.79	

**Table 3:** ICP results for the composition of raw CGs biomass and the best activated carbon.

	Composition, wt%	
Element	CGs Biomass	CGs Activated Carbon
Ca	0.066	0.081
Cu	0.004	0.004
Fe	0.004	0.009
K	0.010	0.042
Mg	0.038	0.044
Mn	0.002	0.002
Na	0.007	0.012
Zn	0.001	0.003

**Table 4:** Textural parameters of selected samples of CGs adsorbents used to adsorb Cu^2+^ from biodiesel.

		Pore Volume, cm^3^/g		
Sample	BET Area, m^2^/g	Micropore	Mesopore	Total
Worst CGs activated carbon—Route No. 3	495	0.240	0.297	0.314
Intermediate CGs activated carbon—Route No. 4	37	0.004	0.056	0.050
Best CGs activated carbon—Route No. 5	22	0.003	0.050	0.039

**Table 5:** Calculated parameters for isotherms models for Cu^2+^ adsorption from biodiesel using the best CGs activated carbon.

		Adsorption Temperature, °C	
Model	Parameter	30	40
Langmuir	*K_L_*, L/mg	0.0016	0.0338
	*q_m_*, mg/g	57.7	20.8
	*R* ^2^	0.93	0.95
	Mean error, %	7.3	5.3
Freundlich	*K_F_*, L^1/n^ mg^1−1/n^/g	0.13	2.46
	*_nF_*	1.13	2.52
	*R* ^2^	0.91	0.86
	Mean error, %	8.2	9.4
Sips	*K_S_*, L^n^/mg^n^	0.0012	0.0475
	*q_Sip_*, mg/g	22.8	24.9
	*n_s_*	0.7	1.3
	*R* ^2^	0.96	0.94
	Mean error, %	4.8	4.3

**Table 6:** Breakthrough curve parameters of Cu^2+^ adsorption from biodiesel using fixed-bed columns packed with the best CGs activated carbon and the commercial bone char.

	CGs Activated Carbon		Commercial Bone Char
Parameter	[Cu^2+^]_Feed_ = 10 mg/L	[Cu^2+^]_Feed_ = 40 mg/L	[Cu^2+^]_Feed_ = 10 mg/L
$t_{b}$, min	73	32	66
MTZ	3.60	5.94	4.67
ΔT, min	77	178	134
$r_{F}$	51.60	96.46	65.62
$F_{q}$	0.14	0.38	0.12
*K_TH_*, L/mg·min	8.58	1.55	5.86
*q_bed_*, mg/g	2.07	5.11	1.28
*R* ^2^	0.9952	0.9951	0.9985
Mean error, %	0.71	2.23	0.28

## Data Availability

Data available upon request.

## Supplementary Materials

The following supporting information can be downloaded at: https://www.mdpi.com/article/10.3390/molecules30030483/s1. Table S1. Elemental composition of the CG activated carbons and raw biomass obtained by EDX analysis. Table S2. Elemental composition of the CG activated carbons and raw biomass obtained by XPS analysis. Table S3. XPS calculated areas of the CGs activated carbon before and after Cu^2+^ adsorption.
