# Supplementary material for: Purification of Biodiesel Polluted by Copper Using an Activated Carbon Prepared from Spent Coffee Grounds: Adsorption Property Tailoring, Batch and Packed-Bed Studies
Source: Molecules. 2025 Jan 22;30(3):483. doi: 10.3390/molecules30030483 (PMC11820585; doi:10.3390/molecules30030483)
Supplement: Supplementary file 1 [file molecules-30-00483-s001.zip › molecules-3428954-supplementary.pdf]

SUPPORTING INFORMATION

Calculation of adsorption capacities

Adsorption capacities of CGs activated carbon to remove Cu^{2+} from biodiesel were calculated with the material balance equation:

$$q_{Cu} = \frac{([Cu^{2+}]_0 - [Cu^{2+}]_e)V}{m} \quad \text{(S1)}$$

where $[Cu^{2+}]_0$ and $[Cu^{2+}]_e$ are initial and equilibrium Cu^{2+} concentrations (mg/L), respectively, V is the volume (L) of biodiesel containing Cu^{2+}, and m (g) is the amount of CGs activated carbon.

Equipment and conditions used in the adsorbent characterization

A Thermo Scientific iCE 3000 atomic absorption instrument operated in flame mode with a linear calibration curve was used for copper quantification in all the adsorption experiments. FTIR analysis was performed with a Nicolet IS10 Thermo Scientific spectrophotometer operated within a wavenumber range of 4000–400 cm^{-1}, using KBr pellets to dilute the samples. XRD analysis was performed using an Empyrean diffractometer (Malvern-Panalytical) operated at 45 kV and 40 mA, with Cu radiation (λ = 1.5406 Å) and Bragg-Brentano geometry. The samples were analyzed within an angle range of 10–60 °2θ. ICP analysis was carried out with an iCAP 7000 series device from Thermo Scientific, using argon to generate the plasma. Organic elemental analysis was performed to identify the C, H, and N composition using a LECO Instruments device. WDXRF analysis was carried out with a S8 Tiger instrument from Bruker. A Quanta 3D FEG scanning electron microscope from FEI Company was used for the SEM/EDX analysis. XPS analysis was also performed with a FlexPS-ARPES-E_SPECs model from SPECS, employing monochromatic Al Kα radiation at 1486.68 eV with an inclination of 56.5° relative to the sample normal and a voltage of 15 KV. The charge compensation device was used via Flood Gun for low and high energy, and the spot analysis was also carried out. N_2 adsorption isotherms were measured with a Quadrasorb Evo instrument (Quantachrome Instruments) where the samples were then degassed at 120 °C for 24 h. The pH at point of zero-charge (pH_{pzc}) of the best adsorbent was determined using the methodology of Faria et al. [90] and Kalavathy et al. [91]. For this purpose, 0.1 M sodium chloride (NaCl) solutions were prepared and adjusted to pH in the range 2-7. Then, 0.1 g of the adsorbent was contacted with 50 mL of NaCl solution at a specific pH under constant stirring at 25 °C for 24 h. Subsequently, the adsorbent was removed from the solution and the pH was measured and plotted versus the initial value, the intersection of both lines indicates the pH_{pzc}.

Adsorption models

Several conventional isotherm models were used to correlate the equilibrium data of Cu^{2+} separation from biodiesel using CGs activated carbons. They included the Langmuir, Freundlich and Sips equations. Langmuir model indicates that adsorption occurs via a monolayer on a homogeneous adsorbent surface [81]. This model is defined as follows:

$$q_{Cu,e} = \frac{q_m K_L [Cu^{2+}]_e}{1 + K_L [Cu^{2+}]_e} \quad \text{(S2)}$$

where $q_{Cu,e}$ is the adsorption capacity at equilibrium (mg/g), q_m is the maximum adsorption capacity (mg/g), and K_L is the Langmuir constant (L/mg). On the other hand, the Freundlich model describes the adsorption on heterogeneous surfaces [82]. The model is expressed as follows:

$$q_{Cu,e} = K_F[Cu^{2+}]_e^{\frac{1}{n_F}} \qquad \text{(S3)}$$

where K_F is the Freundlich adsorption constant (($L^{1/n}$ $mg^{1-1/n}$)/g) and n_F is another adjustable parameter. Finally, the Sips model is a combination of the two previous models and contains three fitting parameters [83], which is given by:

$$q_{Cu,e} = \frac{q_m K_s [Cu^{2+}]_e^{\,n_S}}{1 + K_s [Cu^{2+}]_e^{\,n_S}} \qquad \text{(S4)}$$

where q_m is the maximum adsorption capacity (mg/g), K_s (L^n/mg^n) and n_S are the Sips parameters.

Thomas model [87] was used to fit the breakthrough curves. This model assumes that the system has a constant flow rate with no axial or radial dispersion and considers a constant void fraction in the column. In addition, it assumes constant physical properties for both the adsorbent and fluid phases. This can be expressed using the following formula:

$$\frac{[Cu^{2+}]_t}{[Cu^{2+}]_{Feed}} = \frac{1}{1 + \exp((k_{TH}/Q)(q_{Cu,Bed} m_{bed} - [Cu^{2+}]_{Feed} V_{eff}))} \qquad \text{(S5)}$$

where V_{eff} is the purified biodiesel volume (L) and k_{TH} is the Thomas rate constant (L/mg·min).

Results of CGs activated carbon characterization

Table S1. Elemental composition of the CG activated carbons and raw biomass obtained by EDX analysis.

Sample	*Composition, wt%*								
	C	*O*	*F*	*Mg*	*Si*	*S*	*K*	*Ca*	*Cu*
Best CGs activated carbon – Route No. 5	74.25	24.81	-	0.15	0.18	0.10	0.24	0.13	0.20
Worst CGs activated carbon – Route No. 3	75.35	21.75	0.67	0.17	1.67	0.08	0.31	0.16	0.19
Intermediate CGs activated carbon – Route No. 4	74.49	24.49	-	0.18	0.09	0.16	0.27	0.17	0.28
Raw CGs	64.88	34.56	-	0.11	0.09	0.16	0.06	0.20	0.35
CGs char	48.82	28.42	-	0.22	-	0.12	22.45	-	-
Best CGs activated carbon + Cu^{2+}	74.58	23.59	-	0.17	0.15	0.11	0.18	0.12	1.09

Table S2. Elemental composition of the CG activated carbons and raw biomass obtained by XPS analysis.

Sample	*Composition, wt%*					
	C	*N*	*O*	*Ca*	*Si*	*Cu*
Best CGs activated carbon – Route No. 5	69.26	5.06	25.07	0.60	-	-
Best CGs activated carbon – Route No. 5 + Cu^{2+}	69.56	4.79	22.87	-	1.01	1.76

Table S3. XPS calculated areas of the CGs activated carbon before and after Cu^{2+} adsorption.

	CGs activated carbon - Route No. 5	*CGs activated carbon Route No. 5 + Cu^{2+}*
Peak	*Area*	*Area*
C 1s	101,007.77	110,324.31
N 1s	10,578.82	10,894.02
O 1s	67,868.33	67,381.25
Cu 2p	---	4,948.89
